# Labium Majus Ectopic Pregnancy: Diagnostic and Therapeutic Implications of a Rare Obstetric Phenomenon—A Case Report

**DOI:** 10.1155/crog/6651439

**Published:** 2025-08-25

**Authors:** Katuma Ruvuna, Viateur Hategekimana, Jean Bizimana Kalibushi, Richard Kabuseba Kabuyanga, Théophile Barhwamire Kabesha, Zacharie Kibendelwa Tsongo, Simeon Sibomana, Vincent Kalumire Cubaka, Stephen Rulisa, Jean-Baptiste Sakatolo Zambèze Kakoma

**Affiliations:** ^1^Department of Obstetrics and Gynecology, University of Rwanda, Kigali, Rwanda; ^2^Département de gynécologie obstétrique, Université de Goma, Goma, Democratic Republic of the Congo; ^3^Département d'ophtalmologie, Université Officielle de Bukavu, Bukavu, Democratic Republic of the Congo; ^4^Département de Médecine Interne, Université de Kisangani, Kisangani, Democratic Republic of the Congo; ^5^Policy and Applied Research, High Lands Centre of Leadership for Development, Kigali, Rwanda; ^6^Division of Knowledge and Social Medicine, Partners in Health/Inshuti Mu Buzima, Kigali, Rwanda; ^7^Department of Community Health and Social Medicine, University of Global Health Equity, Kigali, Rwanda; ^8^Département de gynécologie obstétrique, Université de Lubumbashi, Lubumbashi, Democratic Republic of the Congo; ^9^École de sante publique, Université de Lubumbashi, Lubumbashi, Democratic Republic of the Congo

**Keywords:** ectopic pregnancy, labium majus, marsupialization, nontubal ectopic pregnancy, vulvar swelling

## Abstract

Ectopic pregnancy occurs when a fertilized ovum implants outside the uterine cavity, most commonly in the fallopian tube. While rare, ectopic pregnancies can also occur in atypical locations such as the ovary, cervix, abdominal cavity, or broad ligament, often posing significant diagnostic and therapeutic challenges. We report a highly unusual case of ectopic pregnancy implanted in the left labium majus, a site not previously documented in the literature. A 20-year-old woman presented with abdominal pain, vulvar swelling, and amenorrhea of approximately 4 months. Initial imaging and clinical evaluation did not reveal the ectopic implantation. Diagnosis was made intraoperatively during marsupialization of the vulvar swelling, which revealed a macerated fetus within the left labium majus. This case highlights the importance of maintaining a high index of suspicion for rare ectopic pregnancy sites in patients presenting with unexplained vulvar symptoms and a positive pregnancy test—particularly when imaging is inconclusive. It underscores the critical role of prompt surgical exploration in preventing life-threatening complications and contributes to expanding the clinical spectrum of ectopic pregnancy presentations.

## 1. Introduction

Ectopic pregnancy occurs when a fertilized ovum implants outside the uterine cavity, most commonly in the fallopian tube—a condition referred to as tubal pregnancy [1]. Ectopic pregnancies account for approximately 1%–2% of all pregnancies and remain a leading cause of first-trimester maternal morbidity and mortality, primarily due to complications such as rupture and intra-abdominal hemorrhage [2, 3]. While the majority are tubal, other less common implantation sites include the ovary, cervix, abdomen, interstitial region, and broad ligament [4]. These atypical locations often pose significant diagnostic and therapeutic challenges, particularly when clinical signs are nonspecific or mimic other pelvic or abdominal pathologies [5].

Management of ectopic pregnancy typically involves medical or surgical removal of the gestational tissue, given the nonviability of such pregnancies and their associated health risks if left untreated [6, 7]. In addition to the immediate risks, ectopic pregnancies can have longer term reproductive consequences, including tubal damage, infertility, or increased risk of recurrence [8].

Ectopic implantation within the labium majus is, to our knowledge, unprecedented in the published medical literature. Although ectopic pregnancies involving the abdominal cavity and broad ligament have been reported, no prior case has described implantation in the labium majus [9, 10]. This report addresses a critical gap in the literature by presenting a detailed clinical account of this rare form of ectopic pregnancy. The case underscores the need to consider atypical implantation sites when patients present with vulvar swelling and amenorrhea, especially when conventional diagnoses are excluded. By documenting the presentation, diagnostic process, and management approach, this report is aimed at enhancing clinical awareness and supporting earlier recognition of such rare but potentially serious conditions.

## 2. Case Presentation

### 2.1. History, Symptoms, and Clinical Findings

A 20-year-old woman, gravida 2, para 1, with a history of cesarean section, presented to the Gynecology and Obstetrics Department at Kigali University Teaching Hospital (KUTH) with complaints of severe hypogastric pain unrelieved by analgesics and a missed menstrual period for approximately 4 months. She also noted the recent onset of left labial swelling, which prompted her visit. There was no history of fainting or signs of acute abdomen, and her vital signs were stable on admission. Given the combination of prolonged amenorrhea, persistent pelvic pain, and newly developed vulvar swelling, the patient was admitted for thorough evaluation and management.

On physical examination, the patient weighed 55 kg, with a blood pressure of 118/64 mmHg and a pulse rate of 127 bpm. She appeared weak but alert, with normal conjunctival coloration and no signs of jaundice or fever. Cardiovascular examination revealed a symmetrical chest with clear heart sounds. Abdominal examination indicated a soft but tender lower abdomen, with significant tenderness localized to the left lower quadrant. The left labium majus was swollen and tender, with fluid accumulation suggestive of an abscess or bartholinitis. Pelvic examination revealed a normal, closed cervix on digital examination. Despite these findings, her overall condition was relatively stable.

### 2.2. Diagnostic Assessment and Therapeutic Intervention

A urine pregnancy test confirmed a positive result. Ultrasound imaging (not archived) revealed an empty uterus and a well-defined mass adjacent to it, measuring 34.9 × 32.7 cm, containing high-density material with no free fluid in the pouch of Douglas. Due to these ambiguous findings, further evaluation was warranted.

Preoperative workup included complete blood count (CBC), blood grouping and Rhesus typing, liver function tests (SGPT and SGOT), kidney function tests (urea and creatinine), and coagulation studies. MRI was not available at the facility at the time of this case, and laparoscopy was not part of the standard clinical practice in the obstetrics and gynecology department due to limitations in equipment and trained personnel. After anesthetic assessment and obtaining informed consent, the patient underwent exploratory laparotomy and marsupialization under general anesthesia.

During surgery, a vertical midline incision was made below the umbilicus. Contrary to the initial ultrasound findings, no mass was identified near the uterus. Instead, tissue consistent with products of conception—specifically placental tissue without identifiable membranes or fetal parts—was discovered within the left round ligament. Histopathological analysis was not performed, as the macroscopic appearance was clearly consistent with placental tissue, and further confirmation was not deemed necessary at the time. Due to diagnostic uncertainty and the enlarged appearance of the uterus, a limited hysterotomy was performed for further exploration. No abnormal findings were observed within the uterine cavity. The tissues within the round ligament were carefully excised, and the uterus was subsequently closed with hysterorrhaphy. The abdominal cavity was closed in layers, and a sterile dressing was applied.

**Figure 1:**
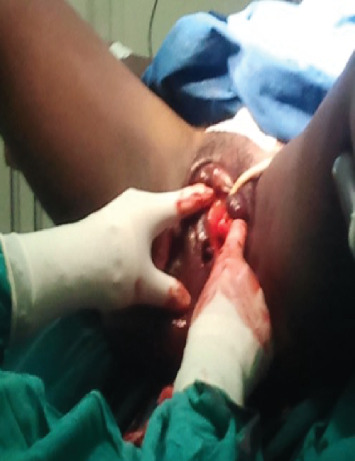
During marsupialization.

**Figure 2:**
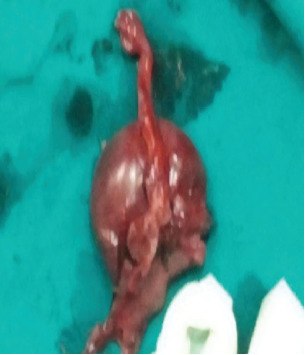
Dead fetus.

**Figure 3:**
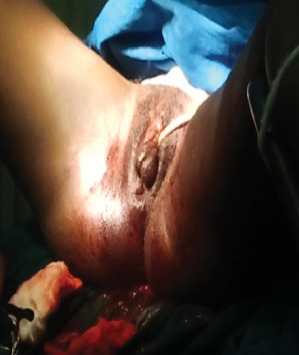
After marsupialization.

With the patient in the lithotomy position, marsupialization of the left labium majus was performed, revealing a macerated, nonviable fetus estimated at approximately 17 weeks gestation, based on gross appearance. Additionally, a Bartholin's abscess was identified. The infected area was thoroughly irrigated and cleaned, and the fetus was carefully removed (Figures 1, 2, and 3). As with the earlier procedure, histopathological examination was not performed, as the findings were macroscopically consistent with a macerated fetus, and further confirmation was not deemed necessary. Postoperative management included broad-spectrum antibiotics (ceftriaxone and metronidazole), intravenous fluids (normal saline and Ringer's lactate), and analgesia with ibuprofen and paracetamol to support the patient's recovery.

## 3. Discussion

This case of ectopic pregnancy in the labium majus is truly unprecedented and pushes clinicians to rethink the typical differential for first-trimester complications. In patients presenting with vulvar swelling and amenorrhea—especially when standard pelvic imaging yields inconclusive results—it becomes vital to consider the possibility of ectopic implantation in rarely affected locations.

Anatomically, the uterus's fibromuscular round ligament extends from the uterine horn through the inguinal canal to the labia majora. In close proximity lies the canal of Nuck, an embryologic remnant akin to the processus vaginalis in males. Though usually obliterated early in life, it may persist in some women as a patent tract leading into the groin or labium. A noteworthy precedent by Noguchi et al. detailed an ectopic pregnancy within a canal of Nuck cyst, suggesting that trophoblastic tissue can indeed migrate through this pathway to implant vulvar tissues [11].

Our hypothesis weaves together this embryological insight with the patient's surgical history. We propose that microdefects at a cesarean scar or uterine horn allowed trophoblastic tissue to access the round ligament, then progress laterally through the inguinal canal and a patent canal of Nuck, ultimately implanting in the labium majus. This mechanism parallels other rare cesarean scar–associated cases, including implantations within the broad ligament or cysts of the canal of Nuck [10–12].

Clinically, this case emphasizes that vulvar masses during pregnancy—which might initially suggest common conditions such as Bartholin's abscess or inguinal hernia—could represent an atypical ectopic implantation. Standard ultrasound may miss such presentations; where available, targeted imaging of the inguinal or labial region—or even MRI—may be warranted when vulvar swelling accompanies amenorrhea. Early surgical exploration under these circumstances can prevent potential hemorrhage and reduce maternal morbidity, consistent with recommendations that stress timely intervention in all ectopic pregnancies [13].

In conclusion, this case is the first documented instance of a labium majus ectopic pregnancy. It broadens the clinical understanding of ectopic gestation sites and underscores the importance of anatomical awareness, surgical history, and thorough evaluation when confronted with atypical vulvar presentations in pregnancy. Clinicians should maintain vigilance for rare implantation routes, prompt diagnosis, and decisive surgical management to avert complications. Moreover, this report highlights the urgent need for continued research into the pathophysiology, diagnostic pathways, and clinical management strategies for such exceptional ectopic pregnancies.

## Data Availability

Data sharing is not applicable to this article as no new data were created or analyzed in this study.
